# Case report: Improving quality of care in Kazakhstan institutions

**DOI:** 10.3389/fpsyg.2022.944729

**Published:** 2022-11-18

**Authors:** Robert B. McCall, Christina J. Groark, Akbota Jappar, Rifkat J. Muhamedrahimov, Oleg I. Palmov, Brandi N. Hawk, Abigail P. Chen, Caitlin F. Spear, Larisa Mason

**Affiliations:** ^1^Office of Child Development, University of Pittsburgh, Pittsburgh, PA, United States; ^2^Ana Yui Foundation, Astana, Kazakhstan; ^3^Department of Psychology, St. Petersburg State University, St. Petersburg, Russia; ^4^CAARE Center, University of California, Davis, Sacramento, CA, United States; ^5^International Assistance Group, Pittsburgh, PA, United States

**Keywords:** community case study, improve caregiving, institutions, infants, development, Kazakhstan

## Abstract

This project is a community case study implemented by local professionals and caregivers to improve the quality of caregiving in two Kazakhstan institutions for infants and toddlers. Local professionals first received comprehensive training by an international team experienced in relevant research-based practices, and then the locals trained institutional staff. Over nearly 2 years, one institution progressively implemented changes in three wards and the other institution in one ward. The changes attempted to make the institution more family-like (e.g., smaller groups and fewer and more consistent caregivers) and caregivers behave more parent-like (e.g., more warm, sensitive, responsive interactions and relationships) without changing nutrition or medical care. Of the 45 children given some exposure to the emerging new wards, 11 experienced the fully revised wards for at least 4 months during their first 2 years of life. They displayed substantial increases in their physical growth, especially those entering in their first year of life, in contrast to the unchanging developmental patterns of 165 children who were reared in the two institutions before the ward changes were made. Physical growth is a commonly used standard of developmental well-being in institutions. Research shows it is sensitive to infants' psychosocial environment, and improvements in physical growth are related to children's cognitive and social-emotional development. Although this pilot community case study had only a few infants fully exposed to the complete ward changes and lacked characteristics of a research experiment, these results are consistent with children's developmental improvements reported in larger scientific studies of similar interventions. This project is an example of how some research-based practices are likely to be implemented in communities in the future. Specifically, it shows that local communities can successfully improve the rearing conditions within institutions, which improve the children's development, and may contribute to the success of their subsequent foster placement and adoption.

## Introduction

Extensive research shows that infants and young children reared in traditional institutions in Russia, Eastern Europe, Latin America, and Asia are drastically delayed in their physical, cognitive, and social-emotional development (i.e., more than a standard deviation below average; Dozier et al., 2012; Berens and Nelson, 2015; McCall and Groark, 2015). Assuming normal distributions for physical growth and behavioral scales, essentially 9 out of 10 children reared in families would be more advanced in their development than the typical resident of these institutions.

Further, extensive research shows that children adopted or fostered from these institutions display higher rates of physical, mental, cognitive, social-emotional, and behavior problems even years after having been placed in these families (Dozier et al., 2012; Berens and Nelson, 2015). Studies also indicate that it is primarily the social-behavioral rearing environment in these institutions that produces these deficiencies, not the genetics, prenatal, and birth circumstances of the children or the medical care and nutrition provided them in the institutions, although these factors have some effect.

Specifically, this conclusion is supported primarily by two studies, among others. In one study, institutionalized children were randomly assigned to professionally-supported foster care vs. remaining in the institution, which controls for a variety of potential selection variables (Nelson et al., 2014). In the other study, all children in an institution experienced fewer and more consistent caregivers who behaved in a more sensitive and responsive manner, which controlled for nutrition, medical care, and other environmental variables (St. Petersburg–USA Orphanage Research Team., 2008). The specially treated children in both studies displayed substantially improved development relative to non-treated comparison children.

For example, the traditional institutional environment typically consists of large wards of homogeneously aged children, separate groups of children with disabilities, with many and changing caregivers who interact with children in a perfunctory, business-like manner. But when this institutional environment is made more family-like and caregiving more parent-like children's physical, cognitive, and social-emotional development improves substantially and some of their long-term problems are reduced (St. Petersburg–USA Orphanage Research Team., 2008; Hermenau et al., 2016; Julian et al., 2019).

## Context

The developmental status of institutionalized infants and toddlers in Kazakhstan in particular is similar to that reported for other countries. A study conducted in 2009–10 under the supervision of the Kazakhstan Academy of Nutrition found that children in 10 institutions for infants and toddlers in the cities of Astana, Almaty, and Karaganda were comparably underdeveloped (Hearst et al., 2014).

In light of this previous research, the Ana Yui Foundation of Kazakhstan started on a path toward welfare reform for vulnerable children in Kazakhstan. An important early step was to demonstrate that local professionals and caregivers could improve the caregiving in two institutions for infants and toddlers. To begin with, Kazakh professionals received comprehensive training by a University of Pittsburgh (USA) and St. Petersburg State University (Russian Federation) team of professionals experienced in research-based practices to improve caregiving in institutions (St. Petersburg–USA Orphanage Research Team., 2008). Then these local professionals trained institutional staff and caregivers, and the institutions implemented aspects of the training according to their own policies, practices, and schedule.

This report is not a traditional research study, but it is a report of the application of research. Specifically, it represents a community-based clinical case study using the train-the-trainer approach (Center for Disease Control Prevention., 2022) to improving children's development and potentially minimizing longer-term problems after adoption or fostering. It is likely that in the future some research-based practices will be implemented in communities using general processes similar to those reported here.

Below we provide brief descriptions of the changes that local professionals made as well as physical growth assessments of children before the changes and of children who experienced the revised environments. This project was considered by the University of Pittsburgh Review Board not to be research but rather an attempt by service agencies to modify their services. Therefore, it was not reviewed.

## Program intervention

### International training

The USA-St. Petersburg team provided initial training that took place on three occasions over 6 months. A total of 25 Kazakh professionals from Astana, Shymkent, and other cities participated. All had some prior training in relevant topics, experience with institutions, and the intention to support children, caregivers, and families in the future.

Sixteen topics were taught covering children of all ages including developmental milestones, developmental risks, responsive caregiving, attachment, parenting, the effects of trauma, mental health, behavioral and psychiatric problems and how to respond to them, coaching and supervision, and changing an institution (based upon the authors' experience reported in St. Petersburg–USA Orphanage Research Team., 2008). A prepared curriculum was used that consisted of written modules, exercises, and discussion topics supplemented by power points, handouts, videos, and instructions on how to train other professionals and caregivers.

### Local training

After the international training, professionals and caregiving staff from each institution were trained. Four participants from the international training trained in seven to eight sessions for a total of 22–29 h ~150 local professionals and caregivers from the two institutions. They selected the topics of mental health, attachment, child development, risk signs, and more respectful caregiver-child interactions.

### Ward changes

Over a span of nearly 2 years, one institution progressively implemented changes in three wards and the other institution did so in one ward. Although the implementation details differed between institutions because of staff resignations (including the director in one institution), renovations required by the city, and other local circumstances, the changes in both institutions similarly emphasized reducing group size, mixing children of different ages within a group, integrating children with and without disabilities in the same group, discontinuing periodic graduations of children to new groups, assigning fewer caregivers per group and having them work more consistently across days, and encouraging caregivers to interact with children in a warm, sensitive, and responsive manner. In short, the changes were an attempt to make the institution more family-like and caregivers behave more parent-like.

Specifically, instead of 9–12 different caregivers serving approximately 12 children and usually different caregivers every day, the revised schedule had six to seven children in a group, ranging in age from 1 month to 4 years of age, including one or two children with disabilities. Children were selected to enter the special wards to create and maintain the diversity of age and disability in the group. Preference was also given to children who were more likely to remain in the institution (i.e., did not have a family likely to take the child back soon).

Children were served by four caregivers during the day and three at night. Although their precise hours changed slightly over time, there were two “primary” daytime caregivers who worked 9–10 h alone on 2 days and then both worked 6 h in non-overlapping shifts on 3 days with 2 days off per week. They were assisted by two nurses, who worked 14 h on two consecutive days and then were off for 2 days, plus two night caregivers. Therefore, children saw one or both “primary” caregivers and two or three of these four daytime caregivers every day. Caregivers were encouraged to interact with children in an engaged, warm, sensitive, and responsive manner. No changes were made in medical care or nutrition. Which specific changes and how and when they were implemented were totally under the control of the institutional administrators, professionals, and caregivers.

## Outcomes

### Children's physical growth in the institutions

The well-being of children within such institutions is typically indexed by the children's physical growth (e.g., height and weight), which is assessed routinely by the institution's pediatricians. Research has demonstrated that children's physical growth is retarded when they are reared in poor psychosocial environments, such as is typically provided by traditional institutions (Hearst et al., 2014), regardless of nutrition and medical care (i.e., the “psychosocial short stature hypothesis;” Skuse et al., 1996). Further, improvements in the psychosocial environment alone have been demonstrated to improve children's physical growth, which in turn is related to improvements in their mental functioning and social-emotional behavior (St. Petersburg–USA Orphanage Research Team., 2008; Johnson et al., 2010). Therefore, physical growth was selected as an index of the potential benefit of these social-behavioral rearing changes for children's development.

### Baseline physical growth before environmental changes

Samples of all children arriving in the two institutions during a specific calendar period of time before any changes were made provided a baseline of 3,795 height and weight measurements over age from 165 different resident children. The measurements were converted to standardized *z* scores according to the WHO Child Growth Standards (2017) which are based on non-institutionalized children. Non-institutionalized children would have a mean *z* score equal to 0.0 with a standard deviation of 1.00.

**Figure 1:**
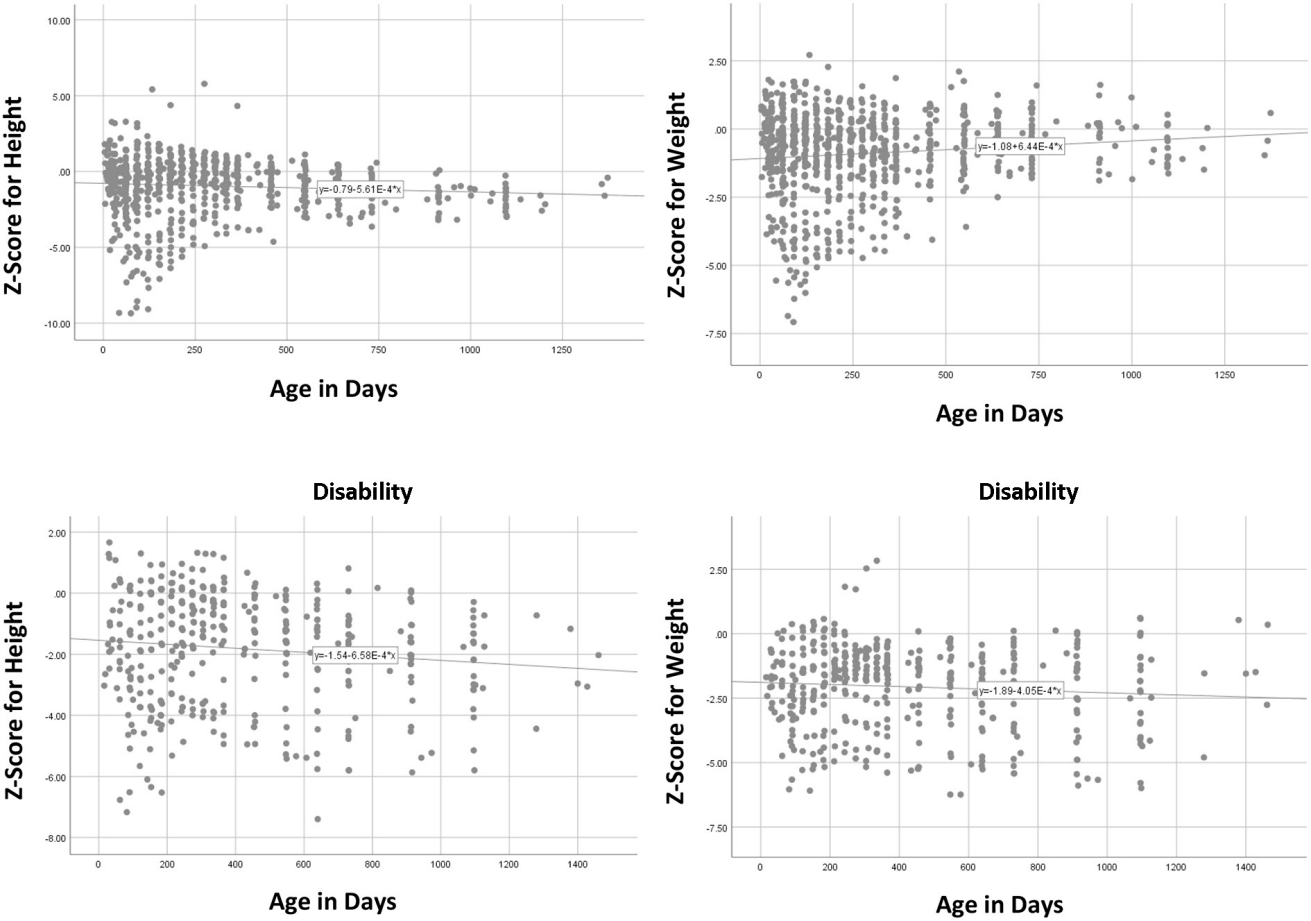
Plots of baseline height **(left)** and weight **(right)** *z* scores across age for typical **(top)** and children with disabilities **(bottom)** before interventions.

These measurements of height (left) and weight (right) are plotted in Figure 1 across age separately for children with (bottom) and without (top) profound disabilities as determined by the institution's pediatricians. Although these data are not strictly longitudinal, it is not likely that selective attrition influenced the developmental trends until the older ages.

These data show that, relative to non-institutionalized family-reared children, institutional residents as a group generally did not improve or decline much in mean relative standing over age. Instead, their growth profiles of standardized height and weight were predominately horizontal straight lines, increasing or decreasing only slightly over age.

Similar to the previous study of institutional children in Kazakhstan (Hearst et al., 2014), the average level of physical growth was approximately one standard deviation below the average (*z* = −1.00) for non-institutional children, lower for children with disabilities. This means that ~84% of non-institutionalized family-reared children would be taller and weigh more than the average institutional child of the same age and gender. As can be seen in the graphs, variability of measures was quite high at young ages, reflecting vast differences in children's personal and environmental circumstances prior to intake, but then children tended toward the institutional mean as they aged.

### The effects of the ward changes on children's physical growth

Forty-five children experienced some form of the intervention. They did not differ from no-treatment baseline children in age, height, and weight *z* scores at intake to the institutions. Because some changes were implemented early and others not until later, of the 45 children in the two institutions who were ever assigned to intervention wards, only 11 experienced the full set of changes for at least 4 months within their first 2 years of life, a period when the effects of the intervention on physical growth have been shown to be most likely because physical growth typically occurs rapidly during this age period (Johnson et al., 2010). Again, although there are few cases, these 11 children were not obviously different from the non-intervention children or the 45 intervention children in terms of age, height, and weight at intake to the institution.

**Figure 2:**
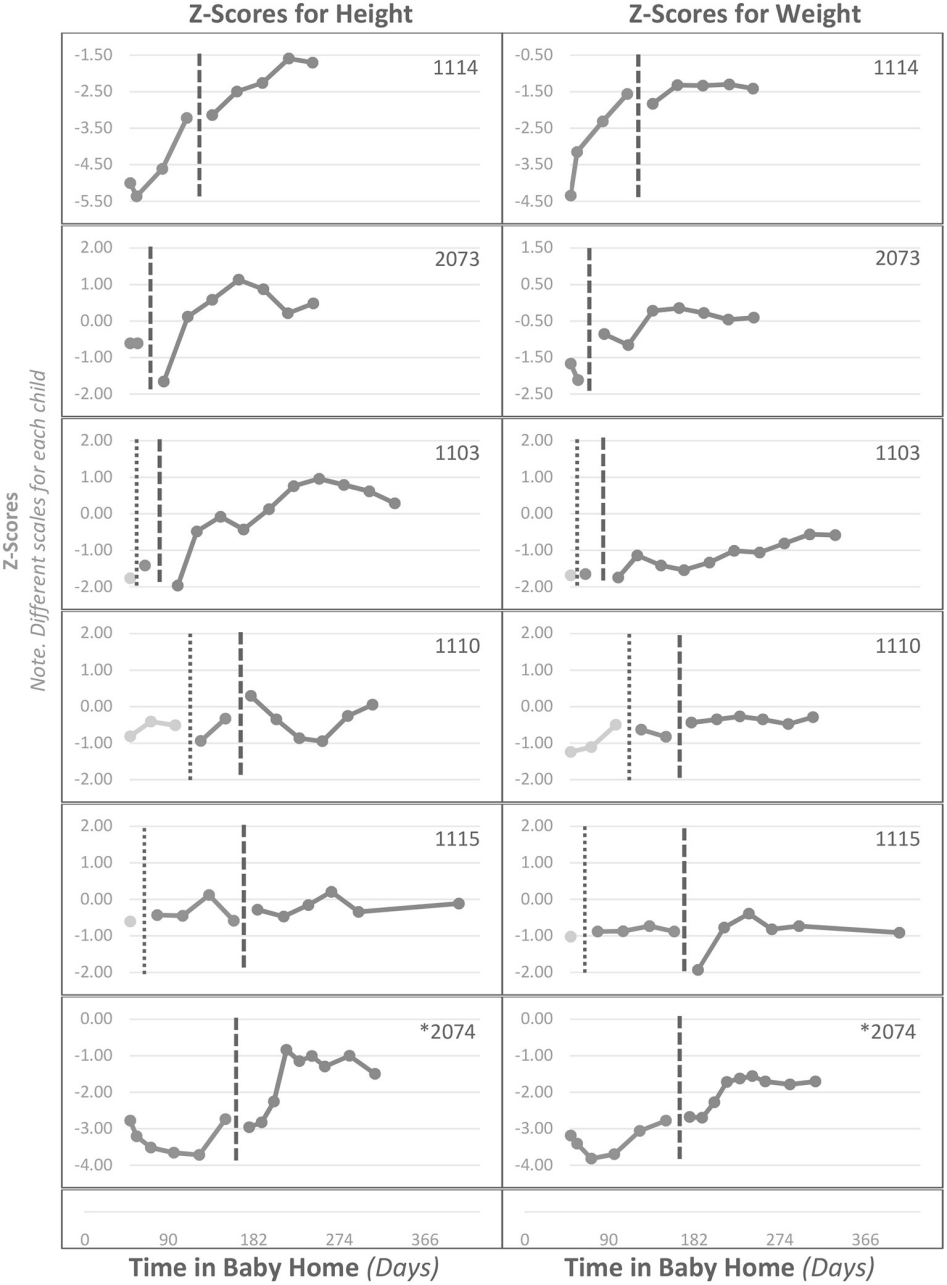
Individual infants' *z* scores across time in the institution (Baby Homes) for height **(left)** and weight **(right)** for infants entering the intervention before 12 months of age and remaining at least 4 months. Data to the left of the vertical dotted line are before entering the intervention, data between the dotted and dashed vertical lines are during an incomplete intervention, and data to the right of the dashed line are during the completed intervention.

Figure 2 presents the growth profiles for six of the 11 infants who entered the newly completed wards within their first 12 months of life and stayed at least 4 months, when the effects of the intervention would be most profound. Assessments made before entering these special wards appear to the left of the doted vertical line, assessments between the dotted and dashed vertical lines were made before the intervention was completed, while those to the right of the dashed vertical line were made after the intervention was completely implemented. The abscissa represents days in residence in the institution. These children entered the institution and began the completed intervention at different ages: #1103 (13, 43 days respectively); #1110 (67,184); #1114 (57, 122), #1115 (89, 256); #2073 (50, 78); #2074 (37,161). Child number ^*^2074 had a disability. Figure 2 shows that the height and weight *z* scores for these six children at the start of the completed intervention were quite varied but were generally within the range of non-intervention children in Figure 2.

Four of the six infants, including one with a disability, showed substantial improvements of 1.5 to 2.0 standard deviations in height and approximately 1 standard deviation in weight. All four of these children entered the completed ward with “stunted” heights or nearly so (*z* =< −2.00), but none ended in stunted condition. One of the other two children irregularly reached new highs in height and regularly in weight, while the other child did not display an increasing profile. Of the six children entering the new wards in their first year of life, four ended with heights at or greater than the average of non-institutionalized family-reared children.

Two of these six children (#1114, #2074) displayed some increase in physical growth before entering the intervention. However, note in Figure 2 that these two children entered the institution extremely underdeveloped (*z* scores between −3.00 and −5.00). As illustrated in Figure 1, such children tend to progressively improve in growth up to the institution average with no special intervention; presumably the traditional institutional environment is better than their pre-intake environment.

Of the five children who entered the completely revised wards after 12 months of age, two showed clear gains, one as much as 2 standard deviations in height and 0.5 in weight. Two other children displayed modest reversals in pre-intervention declines, and one did not show systematic improvement.

## Discussion

Most of children who were sufficiently exposed to the fully revised wards in their early months of life showed substantial increases in their physical growth, including one child with disabilities. Improvements were positive but less profound for children who entered the intervention in their second year of life. Although this case study had only a few infants fully exposed to the ward changes, these results are consistent with larger scientific studies of similar interventions (St. Petersburg–USA Orphanage Research Team., 2008), and they stand in contrast to the generally unchanging growth trends among a large group of untreated residents of these institutions.

Moreover, this result demonstrates that a strictly behavioral change in the caregiving environment can produce improvements in physical growth (St. Petersburg–USA Orphanage Research Team., 2008), and research shows that these improvements in physical growth are accompanied by increases in cognitive and social-emotional measures (St. Petersburg–USA Orphanage Research Team., 2008; Johnson et al., 2010), but these data were not available in this case.

As a community case study, this project lacked numerous procedural and other controls and descriptive details that would characterize a proper scientific demonstration of the intervention's effectiveness. The implementation of changes was left entirely to the discretion of the institutional directors and staff. For example, we do not know the details of how children and caregivers were selected to participate in the intervention (i.e., no random assignment), and we certainly would have liked a larger sample. We had no control over the age of infants when they entered or how long they remained in the revised wards. We know that the wards housed six to seven children ages 1 month to 4 years old most of the time, and that two primary caregivers shared duties during waking hours across the week and one of them was available every day (they were assisted by two other daytime caregivers and two night caregivers). But we have no measurements of caregivers' behavior with the children. Could these and other extraneous factors have contributed to the results?

Of course… Nevertheless, although the two institutions implemented the ward changes somewhat differently and each faced unique challenges and irregularities in their implementation, both created wards with fewer children who were of mixed ages and disability status, fewer and more consistently available caregivers, and more warm, sensitive, and responsive caregiver-child interactions. Further, children who were assigned to the special wards were not obviously different from children who did not experience these wards with respect to their age, height, and weight at intake to the institutions or to the completed intervention. Moreover, the effects on their physical growth occurred over their time in residence, not as a function of their initial values, diminishing concerns about selective sampling. Finally, these basic ward changes and the results on children's growth that we observed were similar to the outcomes of proper and comprehensive scientific studies in which these and other factors were controlled (St. Petersburg–USA Orphanage Research Team., 2008).

## Conclusion

This community-led project illustrates that, with some outside training, local professionals and caregivers can implement changes in institutions' structure, employment patterns, and caregiver behavior that are associated with improvements in children's physical growth, especially in children entering the improved wards in their first year of life. Such improvements have been shown to be related to corresponding improvements in children's mental and social-emotional behavior while residents of the institutions (St. Petersburg–USA Orphanage Research Team., 2008) and years later after placement into families (Julian et al., 2019).

## Data availability statement

The data analyzed in this study is subject to the following licenses/restrictions: the physical growth data on residents of institutions are considered private and access to the data is not permitted by institutions. Inquiries regarding the datasets should be directed to RBM, mccall2@pitt.edu.

## Ethics statement

Ethical review and approval was not required for the study on human participants in accordance with the local legislation and institutional requirements. Written informed consent to participate in this study was provided by the participants' legal guardian/next of kin.

## Author contributions

RBM, CG, AJ, and LM contributed to the design of the project. AJ organized, obtained, and interpreted the data. RJM, CG, and AJ contributed to the drafting and editing of the report. RBM, CG, RJM, OP, and BH contributed to the design and presentation of the training workshops. RBM, AC, and CS contributed to the design and interpretation of the data analysis. All authors contributed to the article and approved the submitted version.

## Funding

This project was supported in part by the Ana Yui Foundation, Astana, Kazakhstan. The Foundation and institutions involved were responsible for selecting and providing local training, designing and implementing the interventions, and collecting the data.

## Conflict of interest

LM was employed by company International Assistance Group. The remaining authors declare that the research was conducted in the absence of any commercial or financial relationships that could be construed as a potential conflict of interest.

## Publisher's note

All claims expressed in this article are solely those of the authors and do not necessarily represent those of their affiliated organizations, or those of the publisher, the editors and the reviewers. Any product that may be evaluated in this article, or claim that may be made by its manufacturer, is not guaranteed or endorsed by the publisher.
